# Impact of the COVID-19 Pandemic on Oncology Clinical Research in Latin America (LACOG 0420)

**DOI:** 10.1200/GO.20.00663

**Published:** 2021-05-06

**Authors:** Aline B. Lara Gongora, Gustavo Werutsky, Denis L. Jardim, Angelica Nogueira-Rodrigues, Carlos H. Barrios, Clarissa Mathias, Fernando Maluf, Rachel Riechelmann, Maurício Fraga, Henry Gomes, William N. William, Camilla A. F. Yamada, Gilberto de Castro Jr, Daniela D. Rosa, Andreia C. de Melo, Raul Sala, Eva Bustamante, Denisse Bretel, Oscar Arrieta, Andrés F. Cardona, Diogo A. Bastos

**Affiliations:** ^1^ Latin American Cooperative Oncology Group (LACOG), Porto Alegre, Brazil; ^2^ Hospital Sírio-Libanês, São Paulo, Brazil; ^3^ Brazilian Group of Gynecologic Oncology (EVA), Belo Horizonte, Brazil; ^4^ Universidade Federal de Minas Gerais, Belo Horizonte, Brazil; ^5^ Núcleo de Oncologia da Bahia (NOB)/Oncoclínicas, Salvador, Brazil; ^6^ Sociedade Brasileira de Oncologia Clínica (SBOC), São Paulo, Brazil; ^7^ Beneficência Portuguesa de São Paulo, São Paulo, Brazil; ^8^ Hospital Israelita Albert Einstein, São Paulo, Brazil; ^9^ A.C. Camargo Cancer Center, São Paulo, Brazil; ^10^ Brazilian Gastrointestinal Tumors Group (GTG), Porto Alegre, Brazil; ^11^ Universidade Federal de Santa Maria (UFSM), Santa Maria, Brazil; ^12^ Instituto Nacional de Enfermedades Neoplasicas (INEN), Lima, Peru; ^13^ Brazilian Group of Thoracic Oncology (GBOT), Porto Alegre, Brazil; ^14^ Instituto do Câncer do Estado de São Paulo (ICESP), São Paulo, Brazil; ^15^ Brazilian Group of Breast Cancer Studies (GBECAM), Porto Alegre, Brazil; ^16^ Hospital Moinhos de Vento, Porto Alegre, Brazil; ^17^ Instituto Nacional de Câncer (INCA), Rio de Janeiro, Brazil; ^18^ Grupo Argentino de Investigación Clínica en Oncología, Rosario, Argentina; ^19^ Chilean Cooperative Group for Oncologic Research (GOCCHI), Santiago, Chile; ^20^ Grupo de Estudios Clínicos Oncológicos Peruano (GECOPERU), Lima, Peru; ^21^ Instituto Nacional de Cancerología, Ciudad del México, México City, México; ^22^ Clinical and Translational Oncology Group, Clínica del Country, Bogotá, Colombia

## Abstract

**PURPOSE:**

COVID-19 has affected cancer care worldwide. Clinical trials are an important alternative for the treatment of oncologic patients, especially in Latin America, where trials can be the only opportunity for some of them to access novel and, sometimes, standard treatments.

**METHODS:**

This was a cross-sectional study, in which a 22-question survey regarding the impact of the COVID-19 pandemic on oncology clinical trials was sent to 350 representatives of research programs in selected Latin American institutions, members of the Latin American Cooperative Oncology Group.

**RESULTS:**

There were 90 research centers participating in the survey, with 70 of them from Brazil. The majority were partly private or fully private (n = 77; 85.6%) and had confirmed COVID-19 cases at the institution (n = 57; 63.3%). Accruals were suspended at least for some studies in 80% (n = 72) of the responses, mostly because of sponsors' decision. Clinical trials' routine was affected by medical visits cancelation, reduction of patients' attendance, reduction of other specialties' availability, and/or alterations on follow-up processes. Formal COVID-19 mitigation policies were adopted in 96.7% of the centers, including remote monitoring and remote site initiation visits, telemedicine visits, reduction of research team workdays or home office, special consent procedures, shipment of oral drugs directly to patients' home, and increase in outpatient diagnostic studies. Importantly, some of these changes were suggested to be part of future oncology clinical trials' routine, particularly the ones regarding remote methods, such as telemedicine.

**CONCLUSION:**

To our knowledge, this was the first survey to evaluate the impact of COVID-19 on Latin American oncology clinical trials. The results are consistent with surveys from other world regions. These findings may endorse improvements in clinical trials' processes and management in the postpandemic period.

## INTRODUCTION

In December 2019, a novel coronavirus named SARS-CoV-2 emerged as a cause of pneumonia in a rising number of patients in China.^1,2^ By March 11, 2020, COVID-19, the illness caused by this virus, was declared a pandemic.^3^ There have been approximately 108 million cases and more than 2.3 million deaths reported because of COVID-19 worldwide. Latin America has been heavily affected, accounting for more than 20 million cases and approximately 643,000 deaths reported by February 16.^4^ Health care systems have been coping with a rising number of severely affected patients demanding hospitalizations, including in intensive care units.^5^ As a consequence, elective visits, procedures, and treatments have been postponed because of COVID-19, with a significant impact on patients with other illnesses.^6^

**CONTEXT**

**Key Objective**
Was there an impact on oncology clinical trials in Latin America during the COVID-19 pandemic?
**Knowledge Generated**
A survey performed in several research centers in Latin America has shown that clinical trials have been deeply affected during the COVID-19 pandemic, with suspension of accruals, reduction of patients' attendance, and other alterations on clinical trials' procedures. Of note, remote methods, such as telemedicine, remote site initiation visits, and remote monitoring, have been widely used.
**Relevance**
The findings may endorse future changes in conduction of oncology clinical trials, especially the incorporation of remote technologies to the research centers' routine.

Patients with cancer may have an increased risk of developing severe events.^7-9^ Active cancer is an independent factor for COVID-19 mortality, and thus, patients who are potential candidates for enrollment in clinical trials could have a higher risk of dying from COVID-19.^10^ Clinical trials represent an important opportunity to access novel treatments for patients with cancer in all stages of the disease, but especially to those who have exhausted standard-of-care treatments and still carry a good performance status.^11^ In Latin American countries, clinical trials are sometimes the only possibility for patients in the public health system to have access to optimal standard-of-care treatments and/or innovative targeted therapies or immunotherapy.^12-14^ Clinical research may be one of the most affected oncology areas during the pandemic, mostly because of the need for frequent in-person visits to the research sites and consequently, more exposure to COVID-19.^15^ At the same time, staff who face patients directly, including research staff in institutions participating in clinical trials, are at higher risk of SARS-CoV-2 infection.^16^ Other services such as study operations and logistics may even face delays that jeopardize the conformity to planned activities at a site level.

To preserve patients' safety, along with compliance with Good Clinical Practice standards, several regulatory agencies published guidance for sponsors and study sites on how to conduct clinical research during the COVID-19 pandemic, which includes evaluation of continuing enrollment of patients onto ongoing trials, remote visits, remote monitoring, and alternative methods of investigational products delivery, among other measures.^17-19^ However, there are local characteristics that should be taken into account when adopting these measures.^20^

Formal policies were adopted in 64% of US research centers, based on a survey recently conducted by ASCO. The Dana-Farber Cancer institute has classified their policies regarding research during the COVID-19 pandemic into levels, which varied from minimal restriction on level 1 (only reduction of on-site staff work) to several restrictions on level 4, including suspension of nontherapeutic research and direct interaction with the patients only if strictly necessary.^21^

There is very limited information on how COVID-19 has been affecting cancer clinical research worldwide, particularly in low- and middle-income countries. To understand the impact of the pandemic on oncology clinical trials, we performed a survey addressed to investigators and research centers across Latin America.

## METHODS

### Study Design and Participants

This was a cross-sectional study that involved a survey sent to investigators and research coordinators of cancer research centers in several countries in Latin American members of the Latin American Cooperative Oncology Group (LACOG). The research protocol was approved by the Ethics committee from Hospital Sírio-Libanês, São Paulo, Brazil, on June 8, 2020. A protocol amendment adding questions to the survey was approved on June 11, 2020. Informed consent was obtained electronically from the person who answered the survey.

### Procedures

The survey consisted of 22 questions regarding COVID-19 and cancer research centers institutional activities during the pandemic. The first questions were about the institution or research center characteristics, such as country of origin, type of health insurance coverage (public *v* private), and COVID-19 cases confirmed at local level. Specific questions regarding research center activities were number of active cancer clinical trials, if formal policies on the research field were developed and which policies were implemented (remote visits, home office, remote monitoring, remote site initiation visits (SIVs), changes on delivery of investigational products, suspension of feasibilities, or others), and if there was a temporary suspension on clinical trials recruitment and the reason for these suspensions in each institution. Questions about the selection procedures, consent, protocol deviations, ethics committee activities, and availability of other teams (such as radiology, surgery, and pathology) in clinical trials were also included in the survey. The last question inquired about the changes implemented during the COVID-19 pandemic that could be continued in the postpandemic scenario. The survey consisted mostly of multiple-choice questions, except for the number of open clinical trials in each institution, which required an exact number as a response. An English version of the survey is available in the Data Supplement.

The survey was hosted on Google Forms and was sent by e-mail and social media (WhatsApp Messenger) for members of LACOG in 350 institutions (250 located in Brazil and 100 in other Latin American countries). Brazilian institutions received the e-mail and survey in Portuguese, and the other Latin American institutions in Spanish. The survey was open to answers from June 26 to September 11, 2020, which included the period of the higher incidence of COVID-19 cases in Latin America so far. To improve the response rate, reminders were sent by e-mail and social media during the period that the survey was open. Initially, the survey was sent to only one representative of each institution. However, because of the low rates of responses, even after sending reminders, the survey was sent to more than one representative of each institution.

The survey results were submitted anonymously, and hence, specific data from each institution or individual cannot be identified.

### Statistical Analysis

Data were gathered and analyzed using the SAS statistical software (version 9.4; SAS Institute, Inc, Cary, NC). The obtained data were submitted to descriptive and analytical statistics. To characterize the epidemiologic profile of the participants, absolute and relative frequencies and measures of central tendency (mean) were applied in the descriptive analysis.

## RESULTS

**FIG 1:**
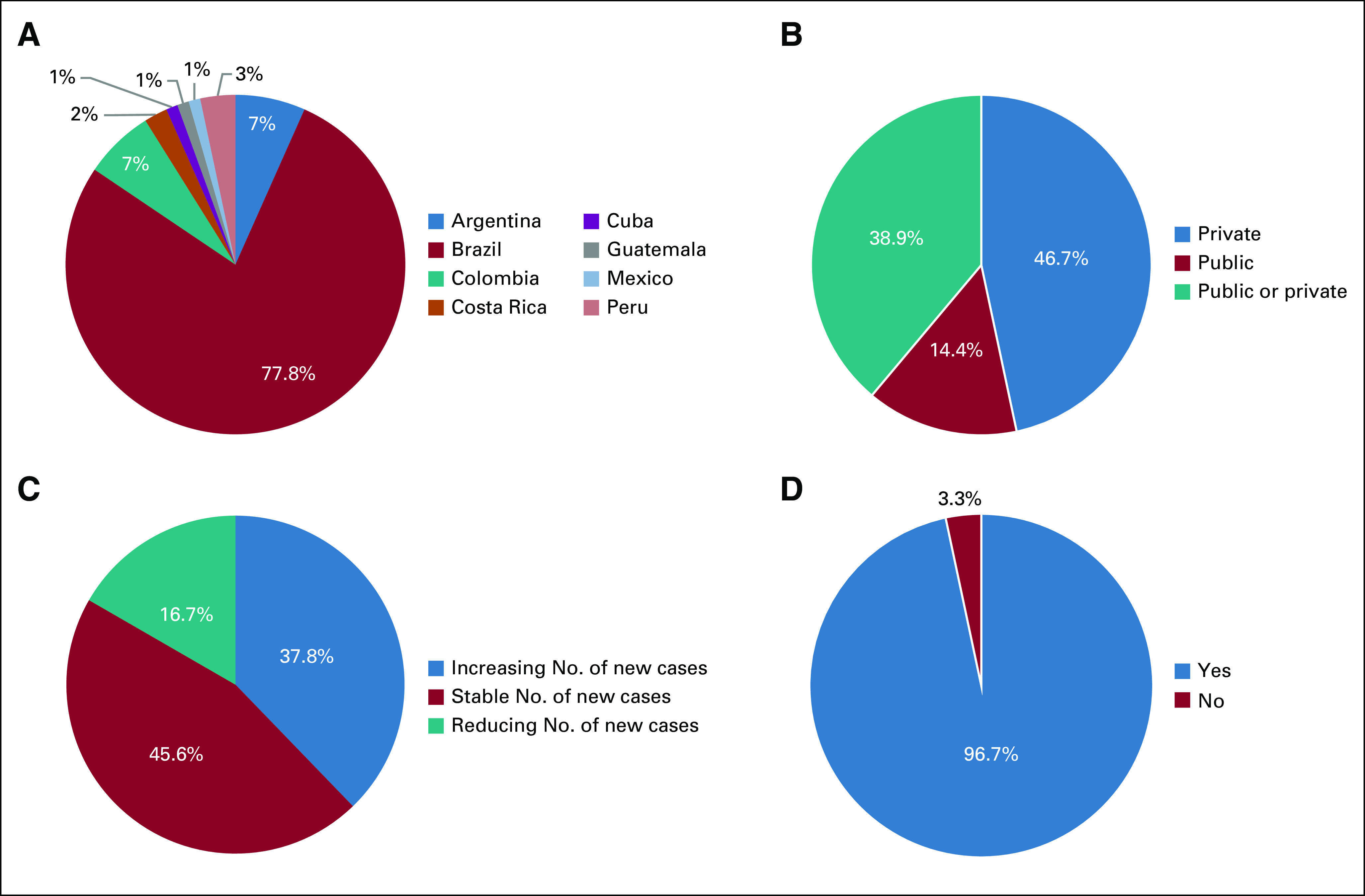
Epidemiology data gathered from LACOG survey responses. (A) Geographic distribution of participating centers. (B) Nature of the participant institutions. (C) Local COVID-19 situation. (D) Existence of policies directed to clinical trials during the COVID-19 pandemic. LACOG, Latin American Cooperative Oncology Group.

Of the 350 questionnaires, 90 responses were obtained for the survey (25%). The majority of responders were from Brazilian research sites (77.8%; Fig 1A). Most of the institutions were partly private or fully private (85.6%; Fig 1B), had confirmed COVID-19 cases (63.3%), and were located in geographic regions with stable or increasing COVID-19 cases (83.4%; Fig 1C) at the time that the survey was conducted. The median number of open oncology or hematology-oncology clinical trials was 10 (1-50) per institution.

**FIG 2:**
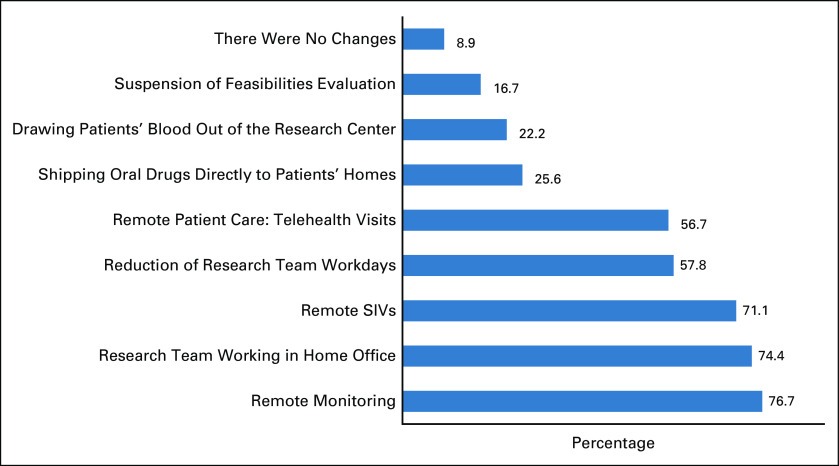
Institutional policies directed to clinical trials during the COVID-19 pandemic. SIV, site initiation visit.

There were specific institutional policies directed to clinical trials during the COVID-19 pandemic in 96.7% of the participant institutions (Fig 1D). Only 8.9% of research centers did not have any change in their processes, 56.7% adopted telemedicine for patient evaluation, 74.4% adopted home office procedures for the research team, and 76.7% had remote monitoring (Fig 2).

**FIG 3:**
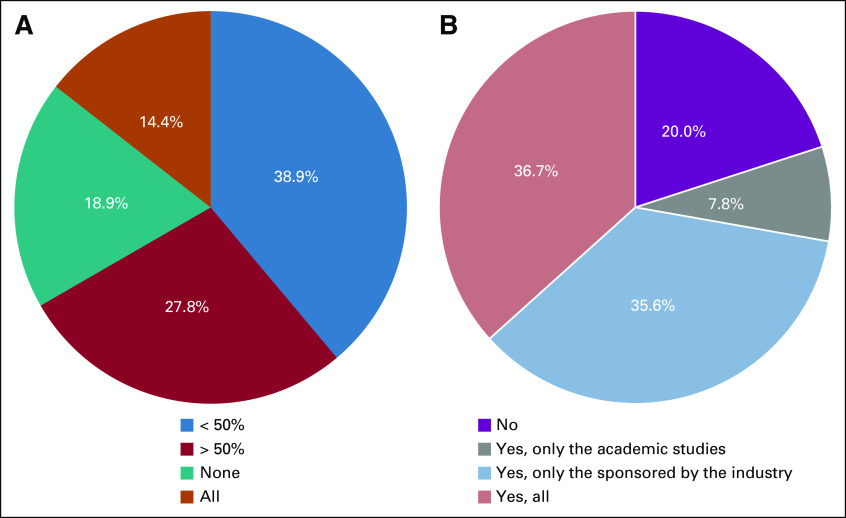
Interruption of oncology clinical trials accrual during the COVID-19 pandemic. (A) Proportion of interruption of accrual in oncology clinical trials in the participant institutions (percentage of trials with interrupted accrual compared with the total of open studies at the research site). (B) Accrual interruption by clinical trial sponsor type.

Temporary interruption of clinical trial accrual occurred in 80% of the participant centers (Fig 3A), mainly because of the sponsors' decision (48.8%). All clinical trial accruals were stopped in 14.4% of the institutions. In 27.8%, < 50% of trials had an interruption, and in 18.9% of the centers, there were no interruptions (Fig 3B). Changes to the screening processes such as prioritizing patients with the largest potential to benefit from therapies, considering the severity of the disease, protocol's safety, protocol efficacy expectation, and other factors were implemented in 27.7% of the participant institutions. Nevertheless, 56.5% continued including all patients fitting the eligibility criteria, regardless of the factors mentioned above. Participants reported that patients treated in their institutions did not show any resistance (unwillingness) (66.6%) to participate in clinical trials during the pandemic, despite the required procedures and number of in-person visits for triage.

**FIG 4:**
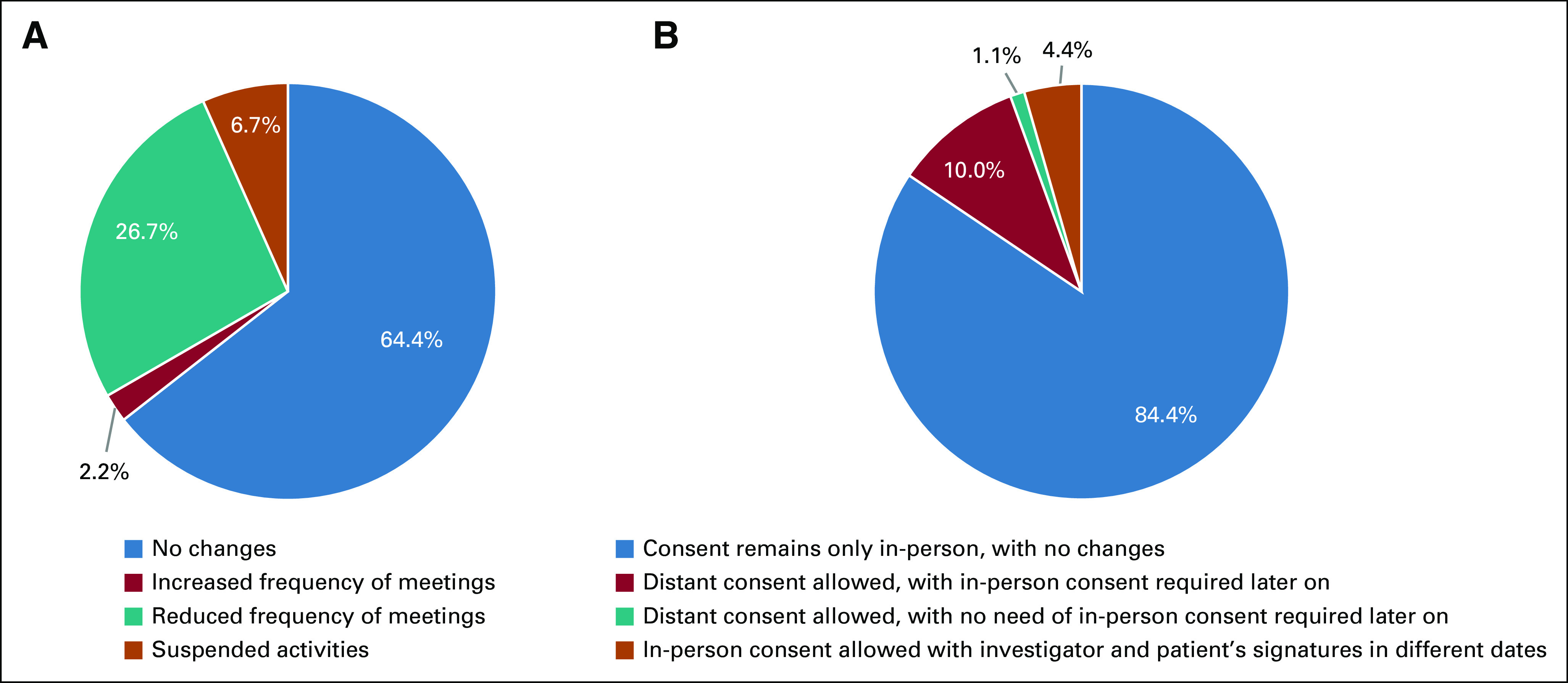
Adjustments in Ethics Committees meetings and Informed Consent procedures in oncology clinical trials during the COVID-19 pandemic. (A) Periodicity of Ethics Committees meetings. (B) Special consent procedures adopted during the COVID-19 pandemic.

Different procedures for consent were adopted only in 15.6% of the institutions, which included electronic consent and investigator's and patients' signatures on different dates (Fig 4B). Frequency of Ethics Committees meetings was not affected in 64.4% (Fig 4A) of the institutions.

**FIG 5:**
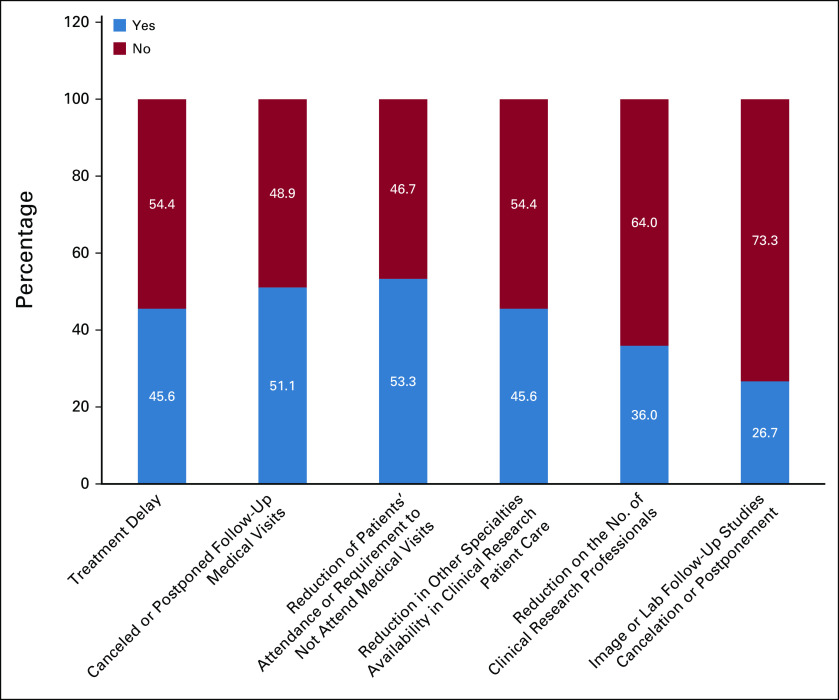
Changes in oncology clinical trials' procedures during the COVID-19 pandemic.

Changes in oncology clinical trials' procedures during the COVID-19 pandemic are described in Figure 5. Treatment delays related to COVID-19 issues were experienced in 45.6% of research sites, which implied in protocol deviations. Image and laboratory facilities were operating normally in most of the research sites (73.3%). Postponement of follow-up image studies, while maintaining a normal laboratory routine, occurred in 20%, and delays of both modalities in 6.6%. Cancelation and/or postponement of medical visits occurred in 51.1%. There was a decrease in patients' attendance or requirement not to attend medical visits in 53.3% of sites. About half (54.4%) of the centers had other medical specialties (radiology, surgery, or pathology) on a normal working schedule. A reduction in the number of research staff during the pandemic was reported by 36% of sites.

**FIG 6:**
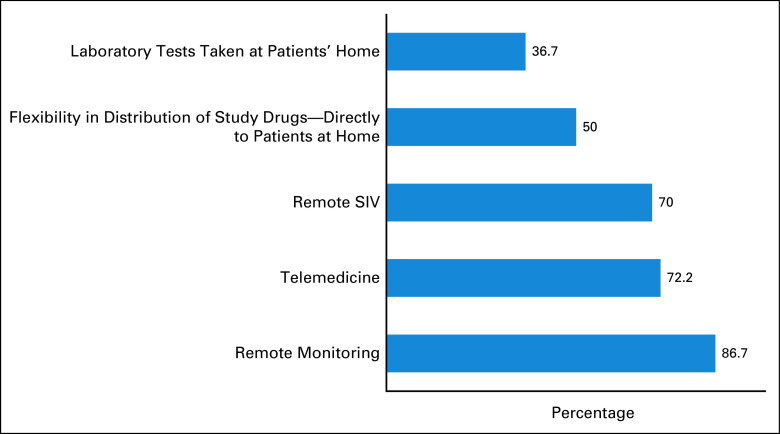
Suggested measures to be adopted on oncology clinical trials in the post-COVID-19 era. SIV, site initiation visit.

Suggested practices to be adopted on oncology clinical trials in the post-COVID-19 pandemic are described in Figure 6. Most respondents recommend remote monitoring (86.7%), implementation of telemedicine (72.2%), and remote SIVs (70%) as possible improvements.

## DISCUSSION

COVID-19 has challenged cancer care.^22^ A pandemic is a rare event that leads to reflections in many aspects of medical care. Patients, caregivers, and health care workers have experienced extreme psychologic distress.^23,24^ COVID-19 has challenged the medical community to implement a much needed, urgent, and immediate response, while dealing with overcrowded hospitals often with not enough supplies to offer the best care to affected patients.^25,26^

Oncology clinical trials continuity has been a subject of great concern during this period. Although trials represent a significant component of oncologic treatment, they come along with a strict routine and very specific procedures,^15,27^ which could be largely affected during the pandemic. ASCO performed a survey among US-based research programs, showing important changes in clinical trials during the pandemic.^28^ To our knowledge, this is the first survey to address the issue in Latin America.

Current results have shown, indeed, a significant impact on the conduct of oncology clinical trials in Latin America. There were specific institutional policies directed to clinical trials during the COVID-19 pandemic in 96.7% of the participant institutions. Of note, there was a difference in the percentage of nonexistence of specific institutional policies compared with percentage of changes in the institutional research processes (3.3% *v* 8.9%). The authors believe that despite having formal policies regarding clinical trials during the COVID-19 pandemic, some institutions did not apply these policies into their clinical trial routine for unknown reasons, justifying this difference between the two responses. The authors speculate that the reasons might be related to local pandemic status (eg, low number of cases), staff conditions (eg, small research site), and number of patients included in ongoing clinical trials (eg, few patients).

Accruals of at least one modality of clinical trials (sponsored or academic) have been suspended according to 80% of the responses. An analysis of a combination of global surveys and interviews of oncology clinical trials investigators and pooled data from IQVIA and clinicaltrials.gov has also demonstrated a negative impact on trials' accruals.^29^ In Europe and the United States, enrollment of new patients remained as usual in only 20% and 14% of institutions, respectively. Asia was less affected, with 60% of their trials recruiting normally.^29^ At Dana-Farber Cancer Institute, there was a statistically significant (*P* = .007) decrease in clinical trials' enrollment as of March 2020 (median = 86 patients), compared with the period between January 2018 and February 2020 (median = 205 patients per month). Moreover, 8.6% of enrolling trials were temporarily or permanently closed and 27.1% were put on hold, mainly because of restrictions on biospecimen collection.^21^ A recent cohort has also examined enrollments, particularly in studies conducted by the SWOG Cancer Research Network, a National Cancer Institute–sponsored National Clinical Trials Network Group. This analysis showed a significant decrease in oncology clinical trials' enrollment over the period that coincided with an increase in COVID-19 cases.^30^ During the LACOG survey period, cases of COVID-19 were stable or increasing in the region of the majority (80%) of sites, which could explain the high rate of accrual interruption. On the other hand, when the survey was performed, COVID-19 has been declared a pandemic for over 3 months. Therefore, almost all institutions had prepared and developed formal policies directed to the management of oncology clinical trials during this period, and accruals' suspension supposedly could be one of these policies.

Modifications on clinical trials' procedures and routines have affected a large proportion (40%) of sites in Latin America. US research centers have also suffered the same problems, with delays on routine services in 38%, decrease in patient's ability or willingness to come to the site, and limited services from other specialties in more than 50% of responses.^28^ During a unique situation as a pandemic, it is hard to control treatment delays and other specialties' availability. Ensuring protocol flexibility to external stressors and having contingency plans while maintaining patients' safety as a priority are some of the strategies that have been adopted.^27^

In a clinical trial routine, it is important to consider patients' requirements and concerns not to attend medical visits. Consequently, we observed an important trend toward the use of remote methods to avoid in-person visits, for both patients and sponsors. More than 50% of sites implemented telemedicine visits, and more than 70% organized remote SIVs and remote monitoring. US-based research programs had a higher percentage of telemedicine visits (87.5%), with similar rates of remote SIVs and remote monitoring.^28^ The lower rates in Latin America might be attributed to limitations such as the access of the population to a good quality internet connection that allows the telemedicine visits, to the absence of legislation regarding telemedicine in some countries, and also to the paucity of telehealth training available across the Latin American countries.^31^ Despite being more than 50%, there was an unexpectedly low rate of patient unattendance or requirement not to attend medical visits, which could also be explained by the patients' limitations associated with the lower rates of telemedicine visits, especially the access to a good quality internet connection, hence the preference for the on-site visits.

The low rates of remote consent could be due to the absence of regulation on this topic by regulatory authorities, and ethics committees be explained by the heterogeneity of ethics committees' procedures across Latin American countries. In addition, some countries demand a national approval to clinical trials' procedures, whereas others allow local evaluation.^32^ During the pandemic, more than 25% of the Ethics Committees had their frequency of meetings reduced, which could lead to a longer time of wait for a remote consent approval. These differences and requirements may difficult the approval of remote consent, added to the difficulty of application, since there is an already mentioned lack of internet access by the general population.

When asked about future suggestions in clinical trials, the use of these tools (telemedicine visits, remote SIVs, and remote monitoring) was the most frequent answers (more than 70% of the responders). The discussion regarding remote methods for patient care was strongly raised during the pandemic,^33^ and there were several suggestions for their adoption.^27,34-36^ In ASCO's survey, telemedicine visits were suggested as an opportunity to improve clinical trials by more than 90% of the respondents, along with remote SIVs and remote monitoring by 70.9% and 64.5%, respectively.^28^ These methods were carried out or planned by the majority of respondents in other survey, endorsing these methods as potential improvements for future clinical trials.^29^ This information could lead to a reassessment of clinical trials' procedures and incorporation of these processes as part of the trials' management. Finally, the incorporation of computer-based tools to help the informed consent process can also be discussed.^37^

Reducing in-person assessments with telehealth visits would allow a higher number of patients to be managed within the research centers' capacity. Moreover, the recruitment of patients who live far away from the research site could be facilitated.^27^ Although telemedicine allows for an adequate patient evaluation in a good proportion of cases, it is important to consider its limitations and recognize as an example, the need for invaluable in-person interactions particularly related to important information collected through physical examination. In Latin America, there might be additional barriers to patients' access to digital communication tools, especially in rural areas. Nevertheless, there have been efforts toward expanding telehealth in Latin-American countries.^38,39^

Changing data monitoring from in-person to remote methods is certainly a challenge since frequently there is an excess of documents to be checked and a few institutions use electronic medical records in Latin America.^40^ However, both remote monitoring and SIVs have been successfully implemented in many research sites during the pandemic, opening a possibility for these procedures to be mostly remote in the future and increasing clinical trials' cost effectiveness.^34,41^

This study has some limitations. The low response rate (25%) should be recognized. As the responses were anonymous, it was not possible to identify multiple responses from the same institution. Despite the fact that the authors believe this is an unlikely scenario, they recognize that this is an important limitation of the study. Also, most of the responders were from Brazilian research sites, which are an important epicenter of the pandemic, possibly prompting an overestimation of the COVID-19 impact in clinical trials.^4^ Nonetheless, Brazil is the leading country in the number of ongoing clinical trials in Latin America.^4,42^ However, the findings of the survey are consistent with data from other reports,^28,29^ showing that this pandemic has largely affected oncology research across world regions.

In conclusion, our survey showed that oncology clinical trials have been significantly affected during the pandemic in Latin America. The impact was greater in clinical trials' accrual, protocol compliance, and monitoring. Incorporation of technology to allow remote protocol procedures has been consistently and largely applied as a risk reducing strategy to protect both patients and site personnel. The results of our survey are consistent with other reports in the literature. Altogether, these results indicate that some of the changes implemented during these unusual COVID-19 times could represent an opportunity for sponsors, investigators, and regulatory agencies to reassess procedures and improve both the patient and staff experience while participating in clinical research.
